# Exploring the Relationship between Urban Quiet Areas and Perceived Restorative Benefits

**DOI:** 10.3390/ijerph16091611

**Published:** 2019-05-08

**Authors:** Sarah R. Payne, Neil Bruce

**Affiliations:** The Urban Institute, Heriot-Watt University, Edinburgh EH14 4AS, UK; N.Bruce@hw.ac.uk

**Keywords:** perceived restoration, public health, quiet area, soundscape, environmental noise, urban park, urban square

## Abstract

To help mitigate the adverse health impacts of environmental noise, European cities are recommended to identify urban quiet areas for preservation. Procedures for identifying urban quiet areas vary across cities and between countries, and little is known of the strength of the salutogenic (health-promoting) benefits they may provide. Taking a multi-site approach, this study examines the potential of three sites as urban quiet areas and their associated health benefits, particularly in relation to perceived restorative benefits. Across three cities in the United Kingdom, an urban garden, urban park, and an urban square had sound pressure levels measured. Responses from 151 visitors to these sites evaluated the place as quiet, calm, and tranquil, and assessed their experience of the place in terms of perceived sounds, its benefits, how it made them feel, and perceived restoration. Depending on the criteria used, the sites varied in their suitability as urban quiet areas, although all provided perceived health benefits. Relationships between sound levels (subjective and objective) and perceived restoration were not linear, with the type of sounds heard and other aspects of the place experience believed to affect the relationship. Building on this work, a future experimental approach based on the study sites is planned to manipulate the multiple variables involved. This will provide a clearer understanding of the relationship between urban quiet areas and perceived restorative benefits.

## 1. Introduction

The relationship between poor environmental acoustics, rising sound levels within urban environments, and its negative impacts on human health have long been documented [1], with the World Health Organization (WHO) concluding “there is overwhelming evidence that exposure to environmental noise has adverse effects on the health of the population” [2] (p. 105). Furthermore, the burden of traffic-related environmental noise in Western Europe has been quantified as “at least one million healthy life years are lost every year” [2] (p. 5). Since then, the WHO has updated its Environmental Noise Guidelines for the European Region exploring both prevention and intervention opportunities for environmental noise to reduce its “public health burden” [3]. Broadly, the guidelines cover transportation noise (e.g., road, rail, air), wind turbine noise, and leisure noise (listening to music through headphones or at various venues). It explores their cognitive effects (annoyance, mental health, cognitive impairments), physical effects (cardiovascular and metabolic, sleep disturbances, hearing impairments, adverse birth outcomes) and impact on overall quality of life and wellbeing.

Examination of environmental noise and its health impacts has predominantly been studied through exposure–response relationships (e.g., [4]). In recent years, a slightly different approach to studying “noise” has developed, which also offers opportunities for examining health impacts through less traditional means than quantified exposure–response relationships. Moreover, the starting point in this approach is a consideration of the acoustic environment in terms of sound rather than noise, recognising that individuals may evaluate sounds differently, thereby only utilising the term noise after public evaluations. This approach is termed soundscapes, which are defined as “acoustic environments as perceived or experienced, and/or understood by a person or people, in context” [5] (p. 1). Soundscape research draws on multiple methods [6], although a recently developed international standard which notes common methodological criteria to be used should increase the ability to compare results across studies [7]. The soundscape approach also enables the consideration of acoustic environments in positive terms, with soundscapes evaluated either positively or negatively, rather than a pure focus on environmental noise.

The potential for positively evaluated acoustic environments aligns with the recommendations from the Environmental Noise Directive (END 2002/49/EC), which despite originating from a noise perspective, require action plans which aim to identify and preserve quiet areas in urban outdoor environments (agglomerations with more than 250,000 inhabitants) to mitigate environmental noise [8]. This infers a potential health value derived from lower sound levels, but only in relation to alleviating the impacts of negative acoustic environments. For example, access to a quiet side of a house may reduce noise annoyance [9] and sleep disturbance [10]. This has resulted in the first of the five WHO environmental noise guiding principles to be “reduce exposure to noise, while conserving quiet areas” [3] (p. 15). Quiet areas, however, may have their own positive beneficial health effects, beyond those of mitigation [11]. For example, they may provide access to hearing other sounds in the city, such as children playing or birds singing, which might perhaps evoke positive feelings, memories, or cognitive improvements.

Currently there is no set definition on what an urban quiet area is, with each European country choosing their own definition and criteria to meet the environmental noise directive [12]. In addition to this, researchers and agencies have also highlighted the importance of other, potentially related, terms for these areas, such as “calm areas” and “tranquil areas” [12,13]. The END, however, suggests the possibility of using averaged day and night sound pressure levels (SPLs) as a way of defining an area as quiet: “an area, …for instance which is not exposed to a value of L_den_ or of another appropriate noise indicator greater than a certain value set by the Member State, from any noise source” [8] (p. L189/14). From this and within the UK, Scotland chose to examine public gardens, open spaces and open land datasets, before applying to those results the specification that the Candidate Quiet Area (CQA) needs to be bigger than 9 hectares, and have L_day_ levels of below 55 dB(A) across 75% of the place [14]. They later adopted the rule that local authorities could also include other areas if justifiable [15]. In contrast in England, CQAs are identified first as outstanding and tranquil local green spaces (or open land if in London), which are “quiet or relatively quiet, and generate significant benefits (in terms of health, wellbeing, and quality of life) for the communities they serve” and could include public gardens, city parks, or urban squares [16] (p. 23). In contrast to Scotland, SPL or land size criteria are not provided, only that the CQAs have a wide appreciation across the city, and as such are accessible. Both countries, however, have chosen to use public green or open spaces as their starting point for defining urban quiet areas “because Local Green Spaces are green areas that have already been identified as demonstrably special to the local community”. Therefore, the beneficial value that these places provide is of importance to their definition.

Indeed, the health benefits of nature and urban green spaces are widely reported across academia, policy, public media, and the World Health Organization [17,18,19]. One of these benefits is psychological restoration, either affectively, cognitively, or physiologically, which helps mitigate against reduced functioning levels (productively, emotionally, or physically) and is related to stress alleviation [20,21]. Generally, natural environments are perceived as more restorative and have higher restorative outcomes, than built environments with limited green or blue (water) features (e.g., [21,22]). Evidenced pathways between natural environments and health benefits include stress reduction, as well as improved air quality, opportunities for physical activity, and increased social connectivity [23]. Reduced sound levels or improved soundscapes may be an additional pathway between the impact of green spaces on health. For example, living near green areas reduces road traffic annoyance and the researchers suggested this could be because they provided a comparatively quieter space nearby (similar to having a quiet side to a house) [24]. Urban park soundscapes are also perceived as having higher levels of restorative components than urban soundscapes predominated by traffic and construction work [25], with perceived restorativeness increasing for more natural urban park soundscapes [26]. Similarly, certain bird sounds are perceived as higher in their restorative potential [27]. However, further research is needed to associate the role of soundscapes as a mechanism for green spaces providing health benefits. Moreover, evidence is so far limited on how to achieve these salutogenic (health-promoting) urban soundscapes with only a few studies examining the positive health effects of soundscapes [24,28].

Therefore, the aim of this project was to take a multi-site approach to explore the restorative and other self-reported health benefits of public urban spaces that could fall into the remit of Candidate Quiet Areas. To meet the aim, first the objective and subjective place characteristics are assessed to determine their suitability as urban “quiet areas”. Secondly, the role of psychological restoration in visiting these places is explored alongside other perceived health benefits gained from being in the place. Finally, the necessity of “quiet” for restoration in urban spaces is examined.

## 2. Methods

Fieldwork was conducted in public urban outdoor places, located in three UK cities in the summer of 2018. Fieldwork consisted of questionnaires with people in the three places and taking acoustic measurements in each place during the same period as the questionnaires.

### 2.1. Environment

Due to their varied geographical locations (South England on the coast, North England in the middle of the country, and Central Scotland on a coast line), the three UK cities chosen for this study were Brighton and Hove, Sheffield, and Edinburgh. Three cities were chosen across the UK as they vary in their architectural style, planning systems (at different stages in identifying candidate quiet areas and using different criteria) and potentially their sound sources (e.g., seagulls in Brighton and Hove). This widens the implications of the findings rather than potentially the results being limited to one city. An urban garden, urban park and urban square were chosen as the study sites. These three types of urban spaces were chosen as they fit within the underlying definitions for CQAs in England (Defra 2018) and the original CQA dataset in Scotland (the Scottish Government 2009). The three chosen places vary in size and are located in city centres, thus accessible to the city population. The choice of places was in part guided by results from a connected study on Project DeStress, where the public identified their quiet areas in these three cities [29]. The Garden was identified as a quiet area by the public and the Park was adjacent to a place identified as a quiet area and was considered part of the same connecting green spaces.

**Figure 1:**
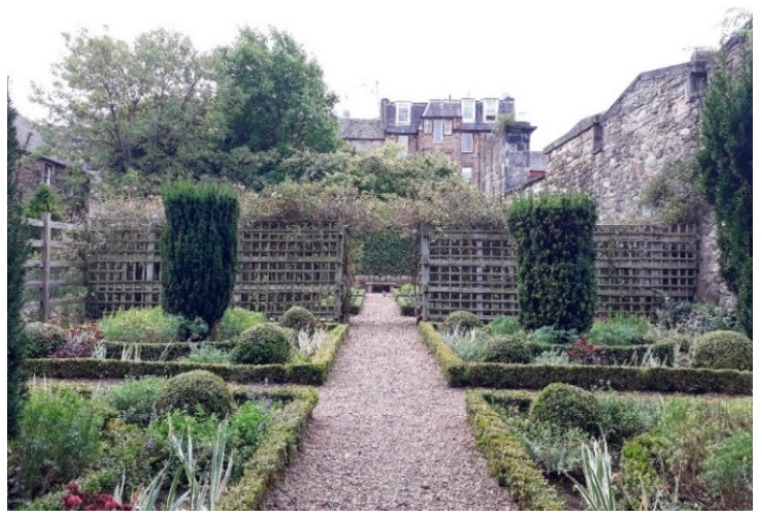
The urban Garden, Dunbar’s Close Garden, Edinburgh, UK, as viewed from the North-West looking South-East within the parterre zone.

The urban gardens in Edinburgh was Dunbar’s Close Garden (from herein referred to as the Garden) in the city centre, only 0.19 Ha and just off the famous Royal Mile. The Garden (Figure 1) is landscaped to replicate a 17th century garden, and has distinctive zones consisting of a gravel courtyard with a tree in the middle surrounded by low shrub patterns, two parterres (symmetrically patterned planting beds), yew bush square borders, a square lawn, a wild area, and a long flower border. The gravel path can be followed on a circular loop through the garden. One or two stone or wooden benches are located in each zone except the long border. To the east of the long border are single-storey and multi-storey residential buildings and to the south-east another gated archway through to the Royal Mile. At the north of the garden behind the square lawn there is no exit and a steep slope down which is covered in trees hiding the high-rise apartment blocks. On the west is a large churchyard behind a high stonewall, while the entrance to the garden is off the royal mile down an archway (close) cut into a large five storey stone building which shields the garden from the cars, coaches, buses and pedestrians on the Royal Mile.

**Figure 2:**
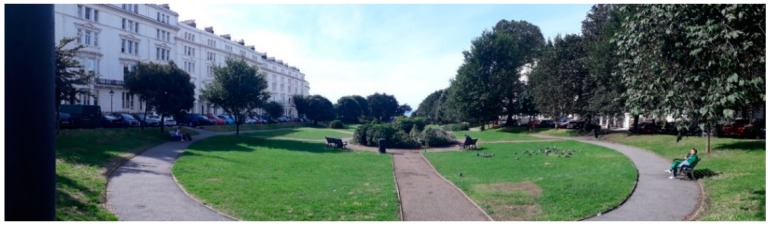
The urban Park, Palmeira Lawns, Edinburgh, UK, as viewed from the North looking South.

The urban park in Brighton and Hove was Palmeira Lawns (from here in referred to as the Park), which is a rectangular green park of 0.72 Ha. The park (Figure 2) contains a number of crisscrossing pathways across the mowed lawns, with benches and waste bins frequently placed alongside the paths. The park is largely open mowed grass with a few clusters of shrubs and flowers. Mature trees line the westerly side of the park, with a few more on the easterly side. On the south side there are numerous trees and bushes delimiting the edge of the park, while to the north, the park is completely open to the adjacent street, with a bus stop at the top and a busy minor (B) road. The park has two residential roads running along the east and west, with cars parked all the way along each side of the park. Five-storey white Victorian town houses overlook the park on the west and east side, whilst the sea can be seen in the near distance when looking to the south.

**Figure 3:**
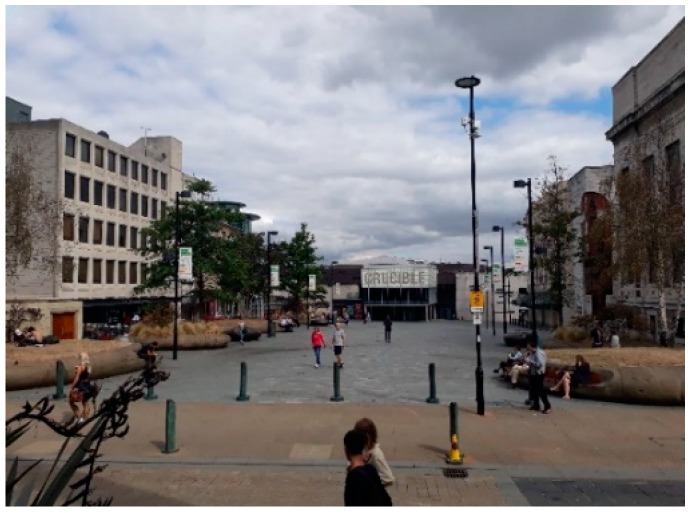
The urban Square, Tudor Square, Sheffield, UK, as viewed from the South-West looking North-East.

The urban square in Sheffield was Tudor Square, in the city centre (from here in referred to as the Square), which is 0.27 Ha. The Square (Figure 3) is known for its cultural activities due to the two theatres and library/gallery flanking two sides of the square (NE and SE). Opposite these stone buildings is a pub and two cafes, both with outside seating areas, with the final side of the square overlooked by a large glass greenhouse with a small cobbled side street (dead end for traffic) in between. The square is paved throughout with 9 stone “pods” of various sizes which contain wooden benches on their sides and are topped by trees or grassy shrubs. There are also three steel pods that can be sat on if not too hot. In two corners, either side of one of the theatres, are street canyons to a side road or a major inner-city carriageway with many bus stops located just behind the theatre.

### 2.2. Participants

Two-hundred-and-sixty-four people in the three places were asked to participate. The positive response rate was 60%, resulting in 159 initial participants. Those under 16 years old (*n* = 1), or whose duration in the place was less than 5 min (*n* = 4) or was unknown (*n* = 3) were removed from all analysis, resulting in a total of 151 participants. There were no significant differences in the response rate depending on the type of day (week or weekend), gender, or if alone or with company (χ^2^ = 0.17, 0.11, 0.79, *p* > 0.05, respectively). There were significant differences in the response rate depending on the place (χ^2^ = 13.24, *p* < 0.001), with more people saying no than yes in the Square (*n* = 70 and 66, respectively), while more people said yes than no in the Garden (*n* = 47 to 18) and Park (*n* = 38 to 17). All participants gave their informed consent for inclusion before they participated in the study. The study was conducted in accordance with the Declaration of Helsinki, and the protocol was approved by the Ethics Committee of the School of Energy, Geoscience, Infrastructure and Society at Heriot-Watt University (Project 316249).

The Park and Square had similar participant demographics. The majority of participants lived in the city (60% and 62% respectively, with 32% and 28% visiting), had a fairly equal gender representation (55% and 52%, female respectively), a median age group of 30 to 39 years olds (varying from 16 years to over 75 years), and a slight urban identity ($\bar{x}$ = 3.92, SD = 1.09 and $\bar{x}$ = 3.51, SD = 1.35 respectively). In contrast, the Garden participants were more frequently visitors to the city (53% compared to 38% living there), were often female participants (72%), had a median age group of 40 to 49 years old (varying from 20 years to over 80 years) and were neutral in their urban to rural identity ($\bar{x}$ = 3.06, SD = 1.37). In total, 81 participants were questioned between 10:00 and 13:59, and 70 participants were questioned between 14:00 and 18:00. Far more participants were questioned on a weekday than at the weekend (*n* = 117 and 34, respectively).

Despite some differences identified above, gender did not significantly differ across the three places (χ = 4.73, *p* > 0.05), and the sample size for age and live/work/visit was too small to be confident of the non-significant results. Urban–rural identity did significantly differ across the three sites with a significantly lower urban identity rating in the Garden than in the Park (F = 4.53, df = 2, *p* = 0.01). Participants answering on the weekend or weekdays did not significantly differ in gender (χ = 0.45, *p* > 0.05), rural–urban identity (F = 0.35, df = 1, *p* > 0.05), or if they lived, worked, or were visiting the city (χ = 2.37, *p* > 0.05), while samples size for age was too small to be confident of the non-significant results.

### 2.3. Measures

To assess the objective acoustic characteristics of the place, 15 min sound pressure level measurements were taken to determine, L_Aeq,15_ (averaged A-weighted level), L_A,max_ (maximum A-weighted level reached), as well as L_A10_, L_A50_, and L_A90_ (L_A10_ level exceeded for 10% of measurement time; L_A50_ statistical average; L_A90_ ambient or background level). Although L_den_ and L_night_ are common measures used when assessing health impacts [3], night-time measurements were not considered necessary for this study, as the study interest was on the health impacts relating to time spent within the places, which are used by the majority of the population in the daytime. To assess the perceived characteristics of the place, participants were asked when “thinking about describing this place, to what extent would you agree with these descriptors”, followed by ten descriptors (Section 3.2.3), and a scale ranging from Disagree (1) to Agree (7). The two authors also made evaluations of the place characteristics using the criteria and parameters listed in the European project Quadmap’s tool for expert assessments [30]. However, neither authors are ecological experts but could make a fair estimation across the three sites.

To understand the use and general experience of the place, participants were first asked two open-ended questions “Why did you come to or pass through this place today?”, “Roughly how long have you been in the place just now?”, followed by closed-ended questions of “Were you with anyone while in this place?” (No, Yes – 1 other, 2 others, more than 2), and “What activity did you do MOST while in this place?” (please tick one). For the activity question, participants were provided with a variety of response options that reflected the Urban, Social, and Aquatic categories previously identified [31] and reflect those utilised in the Monitor of Engagement with the Natural Environment UK surveys [32]. These are depicted in Section 3.1, aside from “energetic physical activities”, “aquatic activities”, and “other” which were not chosen by any participant. The open-ended question ‘please name some sounds you have heard in this place today’ helped determine the soundscape, along with three of the perceived place characteristics (quiet, calm, and tranquil).

To understand the health outcomes from being in the place, participants were asked the open-ended question “What do you think the *benefits* are that you gain from walking through or stopping in this place?”, and “How do you feel in this place?”. In between these, perceived restoration was assessed using existing items [33,34], slightly adapted to prevent an assumption or need for prior fatigue levels (e.g., using “increase” or “improve” rather than “regain” or “renew”). The items asked participants “On a scale from 1 to 7, to what extent do you agree that after having spent some time in this place today, you have now been able to…”. This was followed by eight items, each with a numerical scale alongside ranging from “Disagree 1” through to “7 Agree”. The items consisted of “improve your energy levels”, “increase the ability to concentrate”, “reduce any tension”, “become yourself again”, “ponder over your daily experiences”, “think about your relationships with others”, “think about important issues”, and “see things in a new perspective”. Principal Component Analysis with direct oblimin rotation, and adequate Kaiser’s sample measure (0.89) confirmed a one factor structure (56% variance explained), rather than a separation into two factors representing recovery and reflection. The eight perceived psychological restorative outcomes had high reliability as a set of items that are measuring a similar concept (α = 0.89). This is also the case when the three sites were considered separately (α = 0.91, 0.91, 0.84, for the Garden, Park, and Square, respectively). A perceived restoration score was calculated from the average of participants’ scores for the eight items. Small values reflect low levels of perceived restoration; large values reflect high levels of perceived restoration.

Participants rated their rural or urban identity on a five point scale from rural (1) to urban (5), in line with previous studies [34,35]. They were also asked to indicate if they lived in, worked in, or were a visitor to the city, as well as noting their gender and age.

### 2.4. Equipment

For the acoustic measurements a Casella CEL 246 Sound Level Meter and CEL-120/2 acoustic calibrator (Class 2) were utilised. The CEL 246 is a data logging sound level meter, which stores a time history of the noise levels set to a 1 s interval for the recording period. The CEL 246 also displays the time history as a histogram on the screen and maximum (L_A,max_) and average levels (L_Aeq_). The meter was set to measure A-weighted levels. A windshield was applied to the microphone to reduce wind noise. The CEL246 was mounted on a tripod at 1.5 metres high as necessary for outdoor acoustic measurements [36]. Calibration of the sound level meter to 114db was conducted using the CEL at the beginning of each measurement session.

### 2.5. Procedure

Fieldwork was completed in three sessions (10:00–11:30; 12:30–14:30; 16:00–18:00) on eight days (Tuesday × 3, Wednesday × 3, Saturday × 2), over a three-week period (mid-August 2018 to early September 2018). These days and hours were chosen to ensure the data reflected the range of activities, usage patterns and sonic environments that can occur in and around the three study sites. Additional, ambient sound level measurements were taken on one weekday early morning (05:00 to 07:00) in each city during the fieldwork. During fieldwork hours, temperatures were a chillier 9 to 18 °C ($\bar{x}$ = 15) with wind speeds of 1 to 14mph ($\bar{x}$ = 6) for the Garden, but ranged from 18 to 22 °C ($\bar{x}$ = 20) with wind speeds of 10 to 24 mph ($\bar{x}$ = 16) for the Park, and 18 to 24 °C ($\bar{x}$ = 21) with wind speeds of 2 to 20 mph ($\bar{x}$ = 12) for the Square [37].

For the acoustic measurements, each study site was measured at three to five locations. There were four locations for the Garden (top (S), centre in the Partrees, bottom in the lawn (N), and to the side in the long border (SE)); three locations for the Park (top (N), centre and bottom (S)); and four locations for the Square (top left (N), top right (NE), centre, and bottom (SW)). These selected locations gathered data from distinctly different parts of the sites, to ensure representative measurements for the whole site. Measurements were recorded in each location for each time period, rotating between the locations within each time period. During the measurements, notes were made of any sound events including any interruptions from the public, or weather issues (such as strong gusts of wind). Additional measurements on the pavements by the adjacent major roads for the Garden (the Royal Mile), the Park (busy bus stop street), and the Square (busy bus stop street) were conducted. These were once on a weekday and once at the weekend for the Gardens, once on a weekday for the Park, but at all the same time as the four locations in the Square.

People who had been seen sitting or walking through the places for a period of time were asked to participate in a study about people’s use and experiences of urban outdoor places. Participants were informed that all responses would be treated anonymously, and they could withdraw at any time. Participants were first asked about their general place experience, followed by the benefits gained, their perceived restoration, how they felt, what sounds they had heard, before ending on demographic questions. All questions relating to sound were after questions on the benefits and feelings of being in the place. Upon completion, participants were thanked, debriefed and any questions answered. Questionnaires took an average of 10 min to complete (ranging from 5 to 25 min).

### 2.6. Analysis

Data was collected over three sessions, but at times are analysed here in two sessions, morning/lunchtime (10 am to 13:59 pm) and afternoon/early evening (14:00–18:00). A logarithmic average of the sound pressure level data collected in each of the measurement locations, was calculated using the follow averaging equation where n = number of measurements being averaged.

(1) $$Average\;L_{aeq,15min}=10log_{10}\left[\frac{1}{n}\overset{n}{\underset{i=1}{\sum }}10^{\left(\frac{L_{aeq,15mon}}{10}\right)}\right]$$

The data was averaged for the time periods of 10:00–13:59, 14:00–18:00, and a daily average. To calculate the weekday and weekend average, the daily results were averaged together to form an overall result for the location. The time logged data from the CEL sound level meter enabled calculations of the L_A10_, L_A50_ and L_A90_.

Participants with missing data from items used to calculate a composite score (e.g., missing a response for perceived restoration items, particularly “become yourself again”) are excluded from that individual analysis. Although participants were asked to name one activity, they did the most, some provided multiple answers, and were thus coded as “multiple” activities.

Content Analysis was conducted on all the open-ended questions. The first author initially generated themes by examining responses. Based on frequency counts of these themes, adaptations were made merging or separating some themes. A research assistant then independently coded all responses into the identified themes. Differences were examined and after typing errors were corrected, inter-rater agreement measured by Cohen’s Kappa was 0.74 (*p* < 0.001) for reasons in place, 0.64 (*p* < 0.001) for benefits, 0.83 (*p* < 0.001) for feelings and 0.87 (*p* < 0.001) for sounds heard. These are interpreted as substantial agreement for reasons in place and benefits, and almost perfect agreement for feelings and sounds [38]. Final decisions on the coding were derived by discussing the context of the response and to seek consensus the second author independently coded remaining items that differed, with items finally coded based on the majority response.

## 3. Results

### 3.1. Activity and Social Experience

The Park’ and Square’s participants reported similar activities and social situations (Table 1). The majority of participants main activity was resting, they were by themselves, and had spent around a quarter of an hour in the place (median = 17.5 min and 15 min; both ranging from 5 min to 180 min). The second most frequent activity in both places was doing multiple activities, which were predominantly resting and being social. In contrast, in the Garden although most participants’ main activity had also been resting, a large number had also been doing light physical activities (walking around the gardens). Participants generally spent a much shorter time in the Garden compared to the two other places, ranging from 5 to 60 min (median = 10 min).

Participants not alone in the Park were split between being with 1, 2 or more than 3 other people, while most participants not alone in the Square were only with one other person. A similar proportion of participants were visiting the Garden by themselves as those with one other person, but overall more people were with someone else than alone.

Examining the data across each place and by time of day, the modal activity was still resting in all three places for morning/lunch and afternoon. The modal social situation was being alone, except in the Garden in the afternoon. The median time spent in the place was the same in the morning and afternoon for the Square (15 min), but slightly decreased in the afternoon for the Garden (from 12.5 min to 10 min). In the Park, participants spent on average double the time than of the morning/lunch participants (median is 20 min compared to 10 min), due to the maximum time spent there increasing from 35 min to 180 min.

### 3.2. Suitability of Places as Urban Quiet Areas

To assess the suitability of the three study sites as potential urban quiet areas, the objective acoustic measurements and subjective evaluations of the place and its soundscapes are presented.

#### 3.2.1. Acoustic Measurements

**Figure 4:**
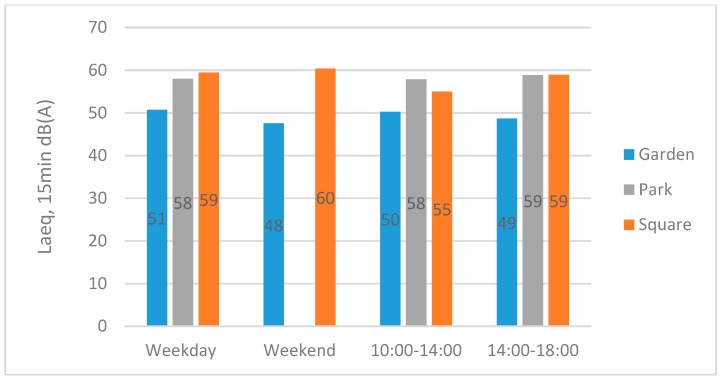
Bar chart of averaged L_Aeq_,_15_ across different types of day (weekday and weekend) and times of day (morning/lunchtime and afternoon) for the three sites.

Across all measurements, the Garden had the lowest averaged sound pressure level (L_Aeq15_) of 49 dB(A), while the Park and Square were substantially higher at 58 dB(A) and 60 dB(A) respectively. There were little differences between averaged sound pressure levels (L_Aeq,15_) for the weekday and weekend in the Garden (51 dB(A) and 48 dB(A) respectively) and in the Square (59 dB(A) and 60 dB(A) respectively) (only weekdays were measured in the Park; Table 2). Similarly, there are little differences between averaged sound pressure levels (L_Aeq15_) for the time of day (morning/lunchtime or afternoon) the measurements were made (Figure 4). In contrast to the daytime variation between the places, the early morning sound levels of each site were much lower and very similar across the three sites (L_Aeq,15_ 52, 52, 54 dB(A) and L_A,max_ 69, 67, 69 dB(A) for the Garden, Park, and Square, respectively). Overall there is little variance within each site during the daytime hours, but greater variation between each site.

The additional road measurements for the Garden averaged at L_Aeq,15_ 66 dB(A), and an L_A,max_ of 80, for the Park they averaged L_Aeq,15_ 61 dB(A), and an L_A,max_ of 70, while the additional main road measurements for the Square averaged at LA_eq15_ 64 dB(A) and an L_A,max_ of 73. For the gardens, this means there was a 17 dB(A) reduction within the Garden compared to on the pavement by the road, while for the Park and Square there was only a 3 and 4 dB(A) reduction respectively compared to near their respective roadsides.

#### 3.2.2. “Expert” Place Characteristic Assessments

Landscape views for the urban Park and Square were similar, with the authors identifying views of the sea, hills, or pleasant architectural structures in three or four directions. In contrast, only two directions from the urban Garden provides a clear landscape view to hills and the adjacent large church. Within the Garden and Park, natural elements were visible in all directions, but were only present on two sides of the Square. The researchers estimated that 95% of the surface area in the Garden was natural compared to 5% artificial materials for the paths, with the natural surfaces consisting of 20% tree canopy, 5% amenity turf, and 70% amenity planting. Similarly, in the Park 85% was estimated to be natural surfaces, but this time consisting of 5% tree canopy, 15% amenity planting, and 65% amenity turf. In contrast, the Square was estimated as only having 10% natural surfaces and 90% artificial, with tree canopy comprising 1%, amenity planting 8%, and amenity turf (on top of the seating pods) 2%. These differences in amount and type of natural surfaces was also reflected in the estimated biodiversity levels, with around 40 habitat types and 6 singing bird species in the Garden, 10 habitat types and four or five singing bird species in the Park, and only eight habitat types and three singing bird species in the Square.

All three sites were regularly maintained and clean, and appeared safe during the daytime, although some drug taking activity was observed in two of the sites. Accessibility was rated high for all three sites, as they were located close to key points, had good transportation networks close by, and were near to residential properties. The Garden had the lowest number of users during the survey hours, with less than 1 user per 9 m^2^ with most areas well used (often as people walked around the place). The larger Park had between 1 and 2 users per 9 m^2^ with more people using the top (near the road and bus stop) and middle of the park, than the more secluded bottom end. The Square was the busiest site, with more than 2 users per 9 m^2^ estimated, with most sitting at the SE end, away from the main road.

#### 3.2.3. Perceived Place Characteristics

A one-way ANOVA test was conducted to assess significant differences between participants’ descriptions of the three places. Significant differences were found for all characteristics, except for the perception of crowded and safety (although the latter was significant, but Bonferroni post-hoc comparisons found no significant differences between each paired place). Depending on the significance results of Levene’s test of homogeneity, Bonferroni or Tamhane’s post-hoc comparisons were examined. Notably the Garden was perceived as significantly quieter, more tranquil, less built up, and slightly less accessible than both the Park and the Square (Table 3). In contrast the Square was perceived as significantly less quiet, less calm, less tranquil, less natural, less biodiverse and a lower feeling of being alone than both the Park and the Square.

Perceived ratings of quiet, calm and tranquil across all three sites had significant moderate positive correlations (*r* = 0.62 for quiet and calm; *r* = 0.59 for quiet and tranquil; and *r* = 0.69 for calm and tranquil; *p* < 0.01). There were also weak to moderate strength positive significant correlations between natural and biodiverse descriptors (*r* = 0.54, *p* < 0.01), natural with quiet, calm and tranquil (*r* = 0.43, 0.47, 0.47, respectively; *p* < 0.01), and natural with alone (*r* = 0.49, *p* < 0.01). Alone also had weak but significant correlations with quiet, calm and tranquil (*r* = 0.45, 0.47, 0.51, respectively; *p* < 0.01), but surprisingly no significant relationship with crowded.

#### 3.2.4. Sounds Heard

Participants named between one and seven sounds they heard while in the place, resulting in 371 sounds, with a similar number named per person in each place (Garden = 2.53 sounds per person, Park = 2.18, Square = 2.56). These were coded into 11 sound categories, ranging from natural sounds (e.g., “*birds*”, “*wind*”), people - other sounds (e.g., “*people*”, “*children*”, where the specifics of the sound being made is unclear and could be vocal or movement based), construction work, traffic sounds, and comments about the sound level (e.g., “*quietness*”) with a few noticing an absence of sounds (e.g., “*no sounds*”) (Table 4). Overall, participants equally mentioned nature (*n* = 85), people’s vocal sounds (*n* = 84; e.g., “*conversations*”, “*people chatting*”, “*laughing*”), and vehicles/traffic (*n* = 77; e.g., “*cars*”, “*traffic*”, “*buses*”), although the number mentioned varied between the three places. In the Garden, the most frequently mentioned sounds were nature, followed by people’s vocal sounds and vehicles/traffic. The most frequently mentioned sounds in the Park were natural sounds, followed by vehicles/traffic. In the Square, the most frequently mentioned sounds were people’s vocal sounds, followed by vehicles/traffic. Dogs were not present in the Garden, people’s footsteps (e.g., “*footsteps on gravel*”, “*people walking by*”) could not be heard in the Park, and neither was distant traffic (e.g., “*cars in the distance*”) due to the proximity of the road, while in the Square no one commented on an absence of sounds.

Pearson Chi square tests with 1 degree of freedom were calculated and assessed with two tailed probability levels for the presence or absence of each sound type being mentioned by a participant, within a place, depending on the time of day (morning/lunch or afternoon) or type of day (weekday or weekend). Only three significant differences were noted for across the time of day, and only two for the type of day (Table 5). In the Garden, vehicles or traffic were more likely reported in the afternoon than in the morning, while recreational or cultural artefacts were more likely reported at the weekend (e.g., an occurrence of music from a residential house) than in the weekday. These were moderate and relatively strong associations respectively as interpreted using existing criteria [39]. In the Park, people, other sounds and dogs were more likely to be reported in the afternoon than morning (moderate associations). While in the Square, recreational or cultural artefacts were more likely heard on a weekday (e.g., the quarterly hour clock bells and pub music) than at the weekend (moderate association).

### 3.3. Psychological Restoration as a Motivator and Perceived Health Outcomes

The reasons for being in the place highlight the role of psychological restoration in motivating people to visit a place. Perceived benefits from visiting the place, how they felt and their perceived restorative outcomes are presented to highlight the perceived health outcomes from visiting the place.

#### 3.3.1. Reasons for Being in the Place

Participants provided 184 reasons why they were in the place (as a few people provided multiple reasons). These were represented by 10 themes, ranging from purposely visiting the place, for the ambient qualities (sun and fresh air, or peace and quiet), because of its location (near to where work/live or another nearby event) or to be able to relax or have food (Table 6). Although the most frequently mentioned reason overall (*n* = 34) was to visit the place (e.g., “*to enjoy the park*”; “*heard about it and was in [travel guide book]*”), the reasons largely differed across the three sites, with purposely visiting the place only for a strong reason for the Garden (*n* = 24). For the Square, its location was the most frequently mentioned reason for being there (*n* = 19), with it commonly used as a stopping point while on the way to another nearby event or place (e.g., “*just been to the library*”, “*come after church*”, “*kill some time before catching train”*). Eating food and drink in the Square was similarly frequently mentioned (*n* = 18), while meeting or spending time with friends/family or just being around other people was also an important reason for a number of participants (*n* = 10; e.g., “*it’s our time together before head off in different directions*”, “*meeting my daughter”*, “*because lonely*”). In the Park, there was a slightly more even distribution of reasons provided, with the most frequently mentioned reason of relaxing or reading accounting for 21% of responses (*n* = 9; *n* = 8 in the Square; and *n =* 5 in the Garden). Interestingly, seeking peace and quiet (e.g., Garden: “*knew it was a nice peaceful place*”, Square: “*nice place to sit and quiet*”) was the 5th most frequently referred to reason (excluding “other”) across all sites (*n* = 14), but was the second most frequently mentioned reason in the Garden (*n* = 9).

#### 3.3.2. Benefits from Being in the Place

Participants named between one and eight benefits gained from being in or walking through the place, resulting in 336 benefits, with the most benefits mentioned in the Square (Table 7). These were coded into 19 types of benefits, ranging from resting, relaxing, feeling good, being in nature, and being with or avoiding people or traffic. Seven types of benefits referred to the place; these benefits described characteristics of the place that they thought were beneficial rather than describing a personal benefit. Overall, participants most commonly mentioned benefit was the place was peaceful, quiet, or calm (*n* = 53; e.g., “*calm place to sit*”, “*quiet”* “*bit of peace and calm from city*” “*relative quiet*”). This was also the most common benefit in the Square and Garden, and the second most common benefit in the Park. In the Garden, some way behind referring to the benefit of the place as being peaceful (*n* = 26), participants frequently referred to gaining a positive feeling (*n* = 14), and that the place felt very different to other places (*n* = 13; e.g., “*switch off from main road*”, “*work in busy environment*”, “*nice feels more comfortable than on streets*”). For the Park, relaxing was the most frequently mentioned benefit (*n* = 14), followed by the place being peaceful (*n* = 9), and then themselves benefiting from gaining a positive feeling (*n* = 8), which included “*its calming*” and “*sense of peace*”. In the Square, being able to rest was the second most common benefit (*n* = 17), only one behind the place being peaceful (*n* = 18). Overall, restorative benefits of resting, relaxing, recovering, and reflecting accounted for 26% of the total responses, but was only 19% of the Garden’s responses, and 31% of the Park’s, and 29% of Square’s.

#### 3.3.3. Feelings in the Place

Some participants already referred to how they felt when they provided the benefits, they gained from being in the place. However, a total of 242 feelings were reported by participants (between one and four per participant) when asked specifically about how they felt in the place (Table 8). A similar number of feelings were reported in the Garden and Square, with fewer in the Park (partly due to the lower sample size). Ten types of feelings were identified, ranging from feeling relaxed, calm, peaceful, happy, good, safe, and supported. A few participants again commented on the place rather than on how they felt (*n* = 15 overall; e.g., “*like it*”, “*nice little garden, cute*”).

Overall, participants most commonly reported feeling was of relaxation (*n* = 83; “*relaxed*”, “*chilled out*”) and this was the most frequently reported feeling in each of the places (*n* = 25, 17 and 41 for the Garden, Park, and Square respectively). In the Garden, a large number of participants reported feeling “*peaceful*”, “*quiet*”, or “*tranquil*” (*n* = 22). In the Park, the second most frequently mentioned benefit was feeling “*happy*” or “great” (*n* = 10), while in the Square it was feeling “*good*” or “*okay*” (*n* = 11) closely followed by feeling “*calm*” (*n* = 10).

#### 3.3.4. Perceived Restoration

Participants, on average only perceived themselves as being slightly restored when leaving the places ($\bar{x}$ = 4.76, SD = 1.31, on a 7-point scale). A one way ANOVA comparing participants’ perceived restoration in the three places, showed significant differences (F(2,129) = 4.64, *p* = 0.011). Bonferroni post-hoc comparisons (homogeneity of variance was not violated) identified participants in the Park ($\bar{x}$ = 5.38, SD = 1.22, *n* = 30) perceived higher levels of restoration than participants in the Garden ($\bar{x}$ = 4.57, SD = 1.41, *n* = 42) and Square ($\bar{x}$ = 4.57, SD = 1.29, *n* = 60).

A one-way ANOVA for the Park showed significant differences between participants’ perceived restoration at different times of day (F(1,29) = 7.31, *p* = 0.012) with higher perceived restoration in the afternoon (Table 9). A between-subjects ANOVA for the Garden and for the Square, showed significant differences between participants’ perceived restoration at different times of day but not between the weekdays and weekend (Table 10) (Garden: F(1,38) = 4.13, *p* = 0.049 for time of day; F(1,38) = 0.71, *p* > 0.05 for type of day; F(1,38) = 0.39, *p* > 0.05 for interaction of time and type of day), (Square: F(1,56) = 5.61, *p* = 0.021 for time of day; F(1,56) = 0.55, *p* > 0.05 for type of day; F(1,56) = 0.05, *p* > 0.05 for interaction of time and type of day). Participants in the Garden had higher perceived restoration in the morning/lunch, while those in the Square had higher perceived restoration in the afternoon, which is the same as for the Park participants.

## 4. Discussion

This study took a multi-site approach to explore the restorative and other self-reported health benefits of public urban spaces that could fall into the remit of a Candidate Quiet Area (CQA). To address the aim, each of the main objectives are discussed in turn. First, objective and subjective place characteristics are assessed to determine the chosen sites suitability as urban “quiet areas”. Second, the role of psychological restoration in visiting these places is explored alongside the perceived health benefits gained from being in the places. Finally, the necessity of “quiet” for restoration in urban places is discussed.

### 4.1. Suitability of Places as Urban Quiet Areas

Given their land type, the study sites of an urban Garden, Park, and Square all have the potential for being defined as an urban quiet area in the UK. If the Scottish guidelines are applied for defining a CQA as having L_day_ levels of below 55 dB(A) across 75% of the place [14], only the Garden would meet this criteria (49 dB(A)). The Garden was substantially quieter than the two other places which were both above the Scottish level; however, all three sites were smaller than their additional recommendation of the place being larger than 9 hectares. For CQA requirements in England, the Square would not be included as only green spaces are permissible outside of London. The Square also only had a small difference between measurements taken on an adjacent road and with the square itself, suggesting it was not “relatively quiet“. Furthermore, participants only slightly rated it above neutral, rather than quiet. In contrast, both the Garden and Park were subjectively rated by their visitors as quiet, and the Garden could be acoustically described as relatively quiet, given the 17 dB(A) difference between the measurements inside and outside of the Garden. A summary table mapping the study sites against English and Scottish CQA criteria [14,16] is presented in Table 11.

Although the sound levels differed between the three places in the daytime, there was no difference in the early morning, confirming the need to take sound level measurements at appropriate times to be able to differentiate between places. Interestingly within each place, there was also very little variation between sound levels taken in the morning/lunchtime and those in the afternoon, nor between weekday and weekend measurements. Given human activities within and around those places (including traffic levels) is expected to vary, the stableness of the measurements is quite surprising.

Sound level measurements alone are not representative soundscape indicators as they do not incorporate perceptions [5,36] and were shown to be unable to distinguish between the variety of sounds heard in this study. Indeed, there was some variation in the types of sounds heard by visitors to the three sites at different times, with significantly more vehicles and traffic sounds heard in the afternoon in the Garden, despite the averaged sound levels remaining similar. Likewise, recreational and cultural artefact sounds were significantly heard more in the weekdays in the Square but more in the Weekend in the Garden. For the Garden, this was largely due to an adjacent residential building playing music when a number of participants were surveyed. Although this did little to the average sound levels, it affected those individuals’ experience of the place, and hence, their assessment of it as quiet and or restorative, possibly due to prior expectation [40]. Thus, it is imperative to ask people about their perception and experience of a place rather than relying on sound level characteristics or criteria chosen by councils, which cannot be agreed upon across cities and countries. This coincides with the European Quadmap project conclusions that quiet area identification should include surveys with the public on their experiences of the place [30].

The sound level also did not relate to the number of sounds heard within the sites. Participants in the Garden and Square noted a similar number per person, suggesting the raised sound levels in the Square were not from one sound source, such as traffic, masking all the other sounds and creating a cacophonic experience but from a combination of sounds. From the freely recalled sounds, eleven categories of perceived sound were identified that were similar to those used in prior urban park studies [34,41], but with people sounds being split into “vocal”, “footsteps/walking” and “other”. Despite the increasing methods for automatic sensor recognition of environmental sounds [42], asking visitors to identify sounds is currently still likely to produce more reliable results, particularly if interested in *perceived* sounds. Moreover, perceived sounds are more important for understanding soundscapes and its impact on people than just the physical presence of a sound source. People’s attendance to the presence of a sound source varies, thus using an automated process of establishing which sounds are present will make it harder to associate health outcomes with experiences, which are influenced by people’s environmental perception.

Associations between objective and subjective sound level measures do exist though [43], and similarly in this study the lower the objectively measured sound level, the subjectively quieter the place was perceived. This suggests sound level measurements can be a good proxy to help identify places (perceived) as *quiet*, but it cannot determine the *quality* of the acoustic environment. To determine the quality, further measures are necessary to indicate the types of sounds that are perceived which importantly shape people’s experience of a place. For example, the process of determining quiet areas in a Greek Island city involved, amongst other processes, informed academics identifying quiet areas and acoustically determining the level of biological and anthropogenic sounds in each of these places [44]. This resulted in proposing two quiet areas, both of which were fairly small, were over L_den_ 55 dB(A) and were the few sites that contained more biological than anthropogenic sounds. Preserving high acoustic quality environments rather than acoustically defined “quiet” environments is increasingly supported [45], suggesting the approach taken by England rather than Scotland to define CQAs may be more appropriate as it incorporates additional criteria than objective physical measurements [14,16]. Therefore, an important aspect highlighted by the England criteria may indeed be the ability of the place to “generate significant benefits (in terms of health, wellbeing and quality of life) for the communities they serve” [16] (p. 23). These issues are explored further in the discussions of the results for the second objective.

The substantial reduction in sound levels for the Garden compared to by the roadside is due to the five-storey thick stone terraced buildings that separates the road from the Garden with only a 2 by 3.5 metre archway (close) providing access. These buildings block the sound waves from directly travelling into the Garden resulting in much lower sound levels, and potentially resulting in the greater perceived quiet and tranquillity compared to the other sites. Prior research has also concluded that secluded backyards and courtyards provide increased tranquillity than urban parks, as the latter tend to be exposed to traffic noise [46], which was the case for this study’s Park. Similarly, it has been recommended that “a good placement of buildings is much more efficient in creating high-quality soundscapes than remediating measures such as placing noise barriers or absorbing materials” [47] (p. 274). Although, the Square also had a few large three-to-five-storey stone buildings between the road and Square, they are completely separate from each other and with a wider gap between the buildings that act as a street canyon. In addition, the Square has very few absorbent surfaces with only a few vegetated raised pods, and the rest of the material is reflective (stone floor, stone or glass building facades). Therefore, the sound of the traffic is reflected into one section of the Square, whilst all other sounds in the Square are also reflected around, increasing the sound levels. The surface material of a place may be just as important as the building facade material in influencing soundscape evaluations. In this study, hearing footsteps in the gravel was frequently commented on in the Garden, whereas no reference to footsteps or the surface material were made in the stone tiled Square, or grass and tarmac pathed Park. One laboratory study has also highlighted the importance of surface material that urban park users walk on in influencing soundscape quality ratings (although conversely they found gravel to be rated significantly lower in quality than grass or wood) [48]. Therefore, in addition to a consideration of the sound sources that are present in a place, the surface material and surrounding façades should also be considered when hoping to create a quiet or high acoustic quality place.

Vegetation and its substrate, is a more absorptive material than stone or glass, with fewer higher frequency sound waves reflected into the surrounding space and reducing sound levels [49]. Indeed, the more vegetation and more biodiverse the study site (as assessed by the authors), the lower the sound levels and the perceived quiet ratings. Similarly, the place with the lower perceived built up ratings (Garden) received the quietest ratings, while the place with the lower perceived (and “expert” evaluated) natural and biodiverse ratings (Square) received the most neutral ratings for perceived quiet. These place differences were again reflected in the most frequently perceived sounds in each place, with natural sounds frequently recalled in the Garden and Park, while people’s vocal sounds were frequently recalled in the Square, reflecting the greater social activities happening there. Although natural sounds were more frequently recalled in the Park, vehicle sounds were the second most frequently recalled sounds, potentially explaining the increased sound levels and lower perceived quiet and tranquillity rating compared to the Garden. Whereas in the Garden, the second most frequently perceived sound was people’s vocal sounds, which are generally at a lower sound level than traffic and were unlikely to reduce perceived quiet and tranquillity levels as much as traffic sounds. In this study, tranquillity ratings varied in line with the differences in perceived vegetation, biodiversity levels and types of sounds heard. Although caution needs to be applied in interpreting these results, given contextual differences across the three sites, the results are supportive of prior research definitions of tranquillity using a mix of physical characteristics (presence of natural views) and sound levels of types of sounds (presence of natural sounds) [13]. It also supports the predominant choice of green spaces as urban quiet areas in England and Scotland [15,16].

Perceived tranquillity ratings were moderately correlated with perceived quiet ratings, suggesting the inclusion of tranquillity in the England criteria [16] is helpful for identifying CQAs. Perceived calm, however, had a stronger correlation with perceived quiet and tranquillity ratings, and “calm” has been suggested by the European Environment Agency [11,12] as an alternative to “quiet” for choosing places to preserve. All three places were rated positively for perceived calm, suggesting that if this alternative criterion is used, they may all be worth protecting, despite the Square not being as natural, nor as quiet. However, perceived calm ratings only significantly separated the Square from the two other places, suggesting additional criteria would still be necessary to help further distinguish between sites. Additional research into public perceptions and use of the terms quiet, calm, and tranquil areas has been conducted in a connected study on Project DeStress [29] and will be fully reported at a later date.

The accessibility of a site is used as CQA criteria by some European nations [12]. In this study, perceived accessibility was rated highly in all three places. Decisions on how accessibility is defined needs consideration though, as alternative objective measurements such as the Euclidean distance (as a crow flies) or by network distance vary in their resulting distances. This has implications on whether an accessible quiet space would be related to beneficial health outcomes, as found when considering nearby (accessible) nature outcomes [19]. A summary table mapping the study sites against alternative CQA criteria is presented in Table 12.

### 4.2. Psychological Restoration as a Motivator and Perceived Health Outcomes

Across all participants, the main reason for being in one of the places was “for the place” itself. To relax or read was the fourth biggest reason (out of 19 categorised reasons), and to have some peace and quiet was the fifth biggest reason overall. This shows the value of restoring and importance of low sound levels for people’s motivation in using these places, although many other reasons also exist for visiting a place. Reasons for the visits also varied substantially across the three places, showing the different benefits the types of spaces can bring. For the Garden, the majority of visitors were tourists who had heard of the place (hence visiting “for the place”), but for those more familiar with the place, they sought the peace and quiet it offered while on a break. This could be interpreted as the place providing a feeling of “Being Away”, which is one of the four components that contribute to a restorative environment in Attention Restoration Theory [20,50]. Indeed, even a travel guide describes it as, “Tucked away at the end of an Old Town close, this walled garden has been laid out in the style of the 17th century, with gravel paths, neatly trimmed shrubs, herbs, flowers and mature trees. A hidden gem, and an oasis of tranquillity amid the bustle of the Royal Mile” [51]. Thus, even the tourists may visit as an opportunity to “be away” from other environment types and hints at the importance of relative quiet or tranquillity (which the Garden was acoustically assessed to have). For the Park, the opportunity to relax was the most frequently mentioned reason for being in the place, with relaxation commonly associated with restoring, restorative components, and little need for restoration [33,52]. The convenience of the place also seemed to be important to many participants as they could stop to rest there as it was near to another event or place they were going to/from, or lived in the surrounding accommodation. This can relate to the place’s “Compatibility” with what an individual wants to do, another component of restorative environments [20,50], as it offers the chance to relax in a convenient location given the time available to them. Similarly, the location of the Square was the main reason for use, particularly for eating food and drink, and then for providing an opportunity to be in a social environment. Thus, the place was compatible with their needs, but this time from a food and social perspective rather than necessarily the need to relax.

The differences in users’ reasons for visiting each place and their potential relationship to components considered necessary in creating a restorative environment, is also reflected by the variance in the perceived restorative outcomes of the users from being in these places. Park respondents had higher perceived restorative outcomes than both the Garden and Square respondents. This reflects the desired intention of the Park users to relax, while many in the Garden were there to “see it” while with a companion, and for the Square it was convenience and the social atmosphere that the users came for, rather than relaxation per se. Being with others can influence optimal restoration levels, as the opportunity to reflect on things can be reduced [53]; therefore, the social experience of the users in the Garden and Park may have lowered their perceived restorative outcomes.

Although perceived restorative outcomes were minimal, as assessed by standard items relating to aspects of Attention Restoration Theory [33,34], a quarter of participants freely stated restorative related terms (rest, relax, recover and reflect). This suggests restorative benefits are gained or at least expected to occur from visiting these places. This was particularly true for the Park and Square respondents (31 and 29% or benefits listed respectively), and less so for the Garden respondents (19%), despite the average perceived restorative outcomes being the same in the Garden and Square. Similarly, feeling relaxed was also the most frequently reported feeling overall from being in the places and for each individual place, accounting for nearly half of participants’ feelings in the Square. In contrast the word restored was used a lot less, although did occur more frequently with Park participants, who reported the higher perceived restorative outcomes. Feeling restored however is not necessarily a term that lay people may use compared to academics familiar with Attention Restoration Theory, thus determining restoration based solely on examining phrases for similar terminology is not necessarily appropriate. Instead, these results indicate that people visited these places in part due to the desire to “restore” and gained feelings that could be associated with restoration. The differences in results between the rated restorative outcomes data and the freely stated benefits and feelings gained from a place, however, does raise issues with the way subjective restoration is currently assessed. Similarities and differences in the terminology used by laypeople and experts for assessing a concept such as restoration and restorative outcomes may need further exploration, as has started with a scale designed to assess the perceived restorativeness of soundscapes [54] which examines lay people’s interpretation of items used to assess perceived restorativeness. Additionally, Attention Restoration Theory and the perceived restorative outcome items focus on cognitive recovery and reflection, but emotional restoration is just as important and can occur rapidly [21]. As people spent less time in the Garden than the other two sites, this may help explain the lower perceived restorative outcomes and reduced use of terms such as relaxing, recovering, and reflecting for benefits, but the increased use of positive feelings and feeling peaceful and calm.

Other health benefits gained from visiting these places included an increase in positive emotions, which can be associated with emotional restoration and improved wellbeing. In general, the language used by participants was positive, referring to increases in, or more of, a positive attribute rather than less of a negative attribute. Unlike much of the environmental noise literature which talks about quiet areas or green spaces as helping to prevent and mitigate from negative feelings [3], the public instead focus on the positive attributes, and salutogenic (health-promoting) benefits. The concept of instoration—which is the improvement of mood or attention without a prior negative or fatigued state above a beneficial value [55]—may also be relevant here as people may not have needed to “recover” (hence the neutral perceived restorative outcomes) but still improved on their already positive, or neutral mood. As prior fatigue levels or emotional states were not assessed beforehand, this study cannot confirm what changes were made and whether their prior emotion was negative or positive, thus caution should be noted in the interpretation of these results in relation to restoration theory. However, the study does highlight the language the public use to describe the perceived health outcomes they gain from visiting these places, which can help future experimental studies assess health outcomes by using terminology with which the public associate.

Many respondents also recognized other positive states, of feeling peaceful, quiet, tranquil and calm. These comments were clearly about how *they* felt from being in the place, rather than a description of the *place*. These terms were the second and third most frequently described feelings after relaxed. Indeed, even the Square which was rated significantly lower in its description of calm than the other two sites, still had “calm” as a frequently used term to describe the users emotional state. This raises questions about using the phraseology of candidate calm areas instead of CQA as suggested by the European Environment Agency [11,12]; should the users *emotional state* of calm be incorporated, or is it only an assessment of the *place* as calm? The same could be applied to the terms “quiet” and “peaceful” which was used both to describe the place (its sound levels) and themselves (emotional state). The complexities of these terms was also explored in a study in Amsterdam where the difference between “quiet” as a mental state, an experienced place and as a situation was examined [56].

The social experience of being with or around other people was also seen as a benefit from visiting these places. Prior research has highlighted the relationship between social relationships and wellbeing and the social value of public urban spaces [19], and this was echoed again in this study. For a few, it helped them feel better and less lonely, thus the busyness of the place was appreciated. A number of people also enjoyed the opportunity to be outdoors in the sunshine, fresh air, and in nature which have all been shown to help people’s wellbeing [19].

Alongside listing benefits to themselves, many people described characteristics of the place. It seems the benefit to the people was the existence of the place itself rather than the benefits it directly derived *them*, or at least they found it easier to describe these benefits. This shows high levels of appreciation of all three places and highlights the value of good quality urban spaces, either involving nature or architectural features, which are well maintained. Appreciated places will subsequently benefit its visitors, and are associated with perceived restorative outcomes directly [34] or via preference [57]. Additionally, the most frequent benefits listed overall was the places being peaceful or calm, and this was before any questions about sound had been mentioned. Even in the Square, which had the highest objective sound level ratings and the lowest subjective rating for quiet, calm, and tranquil, the most frequently mentioned benefit of being in the place was its peacefulness and calmness. Together, these results support the idea that criteria for choosing candidate quiet or calm areas should incorporate both the public perception of the place and objective values [30], and should have a measurable beneficial impact on its users [16]. This currently differs to the process that Scotland use to identify CQAs.

### 4.3. Exploring the Necessity of Quiet for Restoration

Despite the Park and Square having similar sound levels, albeit above the recommended level that Scotland uses to identify CQAs, visitors’ perceived restorative outcomes significantly differed between the two places. Similarly, the Garden’s sound levels were much lower than the Square, yet on average, visitors’ perceived restorative outcomes were the same in these two places. Clearly, with these three sites, there was no clear correlation between sound levels and perceived restorative outcomes. Furthermore, as perceived sound levels varied in line with objective sound levels, there was also no clear relationship between perceived sound levels (quiet) or ratings of calm and tranquillity, with perceived restorative outcomes. Additionally, all places produced frequent feelings of relaxation, and a visitor benefit of feeling at peace and calm. Therefore, the relationship between perceived restoration and subjective and objective assessments of sound level was not a simple one, yet restoration is an important health and wellbeing outcome that is often associated with the benefit of CQAs [11].

The amount of time people spent in the places was a varying factor between the sites, with people spending a shorter time in the Garden than the other sites, and people spending longer in the Park in the afternoon than in the morning. Temporal factors could explain the perceived restorative outcomes between these two sites and between ratings in the Park for the morning and afternoon. The longer someone stays in a restorative environment, the greater the chance for restoration to occur (perhaps to a certain point) and prior fatigue levels will also influence this relationship.

Finally the type of sounds perceived, rather than just the sound levels are important in creating a restorative experience [26]. For example, natural soundscapes are more restorative than urban soundscapes dominated by traffic [25,26,45,58] and individual natural sounds of birds singing are rated highly in their perceived restorative potential [27]. Natural sounds were more frequently recalled in the Garden and Park than in the Square, and fewer vehicle sounds were heard in the morning in the Garden, when higher perceived restorative outcomes were rated. Therefore, despite visitors spending similar amount of times in the Park and Square and experiencing similar sound levels (both subjectively and objectively), the differences in sound types and the level of vegetation and biodiversity between the sites could in part explain the differences in perceived restorative outcomes. Further exploration of all these factors together is necessary, and only through controlling for the differing variables or systematically varying these variables, can a clearer relationship between the soundscape and restoration be established before being applied, if appropriately, as important criteria for identifying candidate quiet or calm areas.

## 5. Conclusions

Typically, much environmental acoustics research and policy focus has been reactive, trying to mitigate negative health outcomes from urban noise exposure. In contrast, preventative health research involving positive health outcomes from exposure to urban sounds is limited. A reconsideration of acoustic environments in terms of soundscapes, rather than environmental noise, helps readdress this imbalance and enables exploring positive health outcomes. Practically, public urban environments need designing in a manner that prevents environmental noise exposure and supports acoustic environments, which are positively evaluated and potentially provide restorative experiences.

Taking a multi-site approach, this work identified the positive benefits people hoped to achieve and felt from visiting an urban garden, park and square, and how a desire for restoration alongside a peaceful, calm and quiet soundscape were valuable for many. These aspects were discussed alongside the study sites match with criteria for identifying urban Candidate Quiet Areas (CQAs) across the United Kingdom. Despite the public perceiving two of the sites as quiet, the local authorities did not recognize them as CQAs, highlighting disparities between criteria used by authorities to identify CQAs and what the public may consider as quiet. Further reflection on suitable CQA criteria may still be necessary, including a greater understanding of public perception of quiet and its value. As part of Project DeStress, an online survey is currently exploring these issues to help identify key physical and social characteristics of places the public identify as quiet, to help determine relevant CQA criteria to use in future UK noise action plans.

The use of three different types of urban public spaces in three different cities, means local contextual variations may have influenced participants’ responses, making it more difficult to draw conclusive results from comparisons across the sites. Similarly, different people were evaluating different sites, thus individual variations (e.g., ages, familiarity with sites) will exist in how people perceived and evaluated the places due to differing preferences and needs, although the averaging of results should help reduce such issues. The sample size in this study does limit the conclusions that can be drawn from the work, as further statistical analysis to control for variance across all the factors could not occur, thus the interpretation of the results is discursive rather than conclusive. For example, in line with existing research, other factors such as the presence of nature, people and available time were likely to influence perceived restoration ratings in this study, but quiet also seemed to be associated with the desire and ability to restore. Future work is planned as part of Project DeStress involving larger sample sizes and virtual simulations of the study sites where variables can be systematically manipulated to establish clearer relationships between sound levels, perceived quiet, sound sources, type of place and the subsequent health benefits, including restoration. Further understanding of the relationships between environmental sound, psychological restoration, and other health benefits will help policy makers and planners to continue their goals in preserving and identifying urban quiet areas that not only help mitigate the adverse health impacts from environmental noise, but also establish salutogenic environments that create healthy, sustainable societies.

## Figures and Tables

**Table 1:** Percentage of participants reporting their main activity whilst in the Garden (*N* = 47 participants), Park (*N* = 38), Square (*N* = 66) and if they were with someone.

		Garden	Park	Square	Overall
**Activity**	Resting	44.7	55.3	62.1	55.0
	Social	4.3	7.9	10.6	7.9
	Cognitive	8.5	10.5	9.1	9.3
	Light Physical	31.9	10.5	1.5	13.2
	Multiple	10.6	15.8	16.7	14.6
**Social Situation**	Alone	40.4	52.6	50.0	47.7
	With 1 person	42.6	21.1	40.9	36.4
	With 2 people	14.9	10.5	6.1	9.9
	With 3 or more	2.1	15.8	3.0	6.0

**Table 2:** Average acoustic measurements at the weekday and weekend in each site.

	Week Day Average					Weekend Average				
	L_Aeq,15_	L_A,max_	L_A10_	L_A50_	L_A90_	L_Aeq,15_	L_A,max_	L_A10_	L_A50_	L_A90_
**Garden**	51	69	59	51	46	48	60	61	52	47
**Park**	58	63	61	57	52	-	-	-	-	-
**Square**	59	74	63	58	54	60	74	62	58	55

**Table 3:** Mean, standard deviations (brackets), and significance testing of participants extent of agreement with each place descriptor (1 = disagree, 7 = agree) in the Garden, Park and Square.

	Garden	Park	Square	Significance
**Quiet**	**6.23 (0.96)**	**5.51 (1.39)**	**4.78 (1.53)**	F(2,144) = 16.21 ***
**Calm**	6.64 (0.57)	6.17 (0.85)	**5.49 (1.2)**	F(2,143) = 20.02 ***
**Tranquil**	**6.34 (0.84)**	**5.42 (1.32)**	**4.22 (1.59)**	F(2,143) = 35.17 ***
**Built Up**	**2.50 (1.55)**	4.11 (1.47)	4.41 (1.63)	F(2,142) = 21.24 *** **^a^**
**Natural**	5.51 (1.57)	5.62 (1.34)	**3.97 (1.69)**	F(2,144) = 18.47 *** **^a^**
**Biodiverse**	4.87 (1.54)	4.89 (1.58)	**3.54 (1.55)**	F(2,144) = 13.38 ***
**Crowded**	2.40 (1.65)	2.86 (1.53)	3.05 (1.54)	F(2,144) = 2.30
**Alone**	4.49 (1.50)	4.03 (1.48)	**3.03 (1.65)**	F(2,142) = 12.28 *** **^a^**
**Accessible**	**5.72 (1.46)**	6.38 (0.86)	6.32 (0.93)	F(2,144) = 4.96 **
**Safe**	6.00 (0.98)	6.03 (0.97)	5.52 (1.26)	F(2,143) = 3.50 *

* *p* < 0.05, ** *p* < 0.01, *** *p* <0.001, **^a^** Tamhane’s post-hoc comparisons were utilised. Bold numericals indicate the place that significantly differs to the two other places.

**Table 4:** Percentage of times each sound category was mentioned by participants whilst in the Garden (*n* = 119 sounds), Park (*n* = 83) and Square (*n* = 169).

	Garden	Park	Square	Overall
Nature	31.9	37.3	9.5	22.9
Dogs	0.0	8.4	0.6	2.2
People - Vocal	18.5	12.0	30.8	22.6
People - Other	1.7	7.2	8.3	5.9
People - Footsteps/Walking	9.2	0.0	3.6	4.6
Recreational or Cultural artefacts	5.9	1.2	10.7	7.0
Mechanical or Construction work	6.7	3.6	10.7	7.8
Vehicles or Traffic	17.6	26.5	20.1	20.8
Distant Traffic	1.7	0.0	3.0	1.9
Sound Level/Acoustics Comment	5.0	1.2	3.0	3.2
Absence of Sounds/Noises	1.7	2.4	0.0	1.1

**Table 5:** Chi square tests of associations between time of day or type of day and sound types heard in the Garden, Park or Square.

Sound Type	Type of Day or Time of Day	Observed Count (Expected Count)			χ^2^	*p*	ϕ_c_
		Garden	Park	Square			
Dogs	Morning		1 (3.7)		5.29 ^a^	0.021 *	0.39
	Afternoon		6 (3.3)				
People - Other	Morning		1 (3.2)		3.85 ^a^	0.050 *	0.34
	Afternoon		5 (2.8)				
Vehicles or Traffic	Morning	9 (12.4)			4.18	0.041 *	0.30
	Afternoon	13 (9.6)					
Recreational or Cultural artefacts	Weekday	1 (4.6)			13.39 ^a^	0.000 ***	0.54
	Weekend	5 (1.4)					
	Weekday			13 (8.6)	7.52	0.006 **	0.35
	Weekend			1 (5.4)			

^a^ Caution should be taken as low expected cell counts. * *p* < 0.05, ** *p* < 0.01 *** *p* < 0.001.

**Table 6:** Percentage of times each type of reason was provided by participants for being in the Garden (*n* = 62 reasons), Park (*n* = 43) and Square (*n* = 79).

	Garden	Park	Square	Overall
For the Place	38.7	9.3	7.6	18.5
Food or Drink	6.5	14.0	22.8	15.2
Event or Place Nearby	4.8	14.0	24.1	15.2
Relax or Read	8.1	20.9	10.1	12.0
Peace and Quiet	14.5	2.3	5.1	7.6
Be with Friends, Family or Around People	1.6	2.3	12.7	6.5
On a Break	11.3	4.7	2.5	6.0
Sun or Fresh Air	8.1	4.7	3.8	5.4
Live or Work in the Place	0.0	11.6	5.1	4.9
Other	6.5	16.3	6.3	8.7

**Table 7:** Percentage of times each type of benefit was experienced whilst in the Garden (*n* = 115 benefits), Park (*n* = 75) and Square (*n* = 146).

	Garden	Park	Square	Total
Rest	6.1	5.3	11.6	8.3
Relax or Read	7.8	18.7	8.9	10.7
Recover	1.7	4.0	4.8	3.6
Think or Reflect	3.5	2.7	3.4	3.3
Food or Drink	0.9	1.3	2.7	1.8
Positive Feeling	12.2	10.7	2.7	7.7
Being in Nature	7.8	4.0	0.0	3.6
Being Outside in Sunshine or fresh Air	5.2	8.0	9.6	7.7
People - Be with or around	0.9	8.0	8.2	5.7
People - Avoid being around	3.5	1.3	0.0	1.5
Traffic - Avoid being by it	0.9	1.3	5.5	3.0
Place - Peaceful/calm	22.6	12.0	12.3	15.8
Place - Beautiful/nice	7.8	4.0	6.8	6.5
Place - Landscape description	5.2	6.7	4.8	5.4
Place - Built environment description	0.9	2.7	4.1	2.7
Place – Clean, tidy and safe	0.9	4.0	4.1	3.0
Place - Location	0.0	1.3	3.4	1.8
Place - Different to another place	11.3	1.3	4.8	6.3
Other	0.9	2.7	2.1	1.8

**Table 8:** Percentage of times each feeling was experienced whilst in the Garden (*n* = 94 feelings), Park (*n* = 53) and Square (*n* = 95).

	Garden	Park	Square	Total
Relaxed /Comfortable	26.6	32.1	43.2	34.3
Peaceful /Quiet /Tranquil	23.4	13.2	7.4	14.9
Calm	14.9	7.5	10.5	11.6
Happy /Great	6.4	18.9	9.5	10.3
Good /Okay /content	6.4	3.8	11.6	7.9
Place Comment	9.6	3.8	4.2	6.2
Restored	1.1	11.3	1.1	3.3
Safe	2.1	1.9	4.2	2.9
Supported (by others)	0.0	1.9	3.2	1.7
Other	9.6	5.7	5.3	7.0

**Table 9:** Participants’ mean perceived restoration for the Park at different times of day.

	Mean	Standard Deviation	*N*
Morning/Lunchtime (10:00 to 13:59)	**4.87**	1.16	16
Afternoon/ Early evening (14:00 to 18:00)	**5.96**	1.05	14
All day (10:00 to 18:00)	5.38	1.22	30

Bold numericals indicate the variables with significant differences.

**Table 10:** Participants’ mean perceived restoration for the Garden and Square at different times and types of day.

Time and Type of Day		Garden			Square		
		Mean	Standard Deviation	*N*	Mean	Standard Deviation	*N*
Morning/ Lunchtime (10:00 to 13:59)	Weekday	4.95	1.15	17	4.36	1.28	22
	Weekend	4.88	0.73	7	4.06	1.03	11
	Total	**4.93**	1.03	24	**4.26**	1.19	33
Afternoon/ Early evening (14:00 to 18:00)	Weekday	4.28	1.79	14	5.03	0.98	15
	Weekend	3.50	1.62	4	4.86	1.27	12
	Total	**4.10**	1.74	18	**4.95**	1.10	27
All day (10:00 to 18:0)	Weekday	4.65	1.49	31	4.63	1.20	37
	Weekend	4.38	1.26	11	4.48	1.21	23
	Total	4.57	1.42	42	4.57	1.19	60

Bold numericals indicate the variables with significant differences.

**Table 11:** Study sites mapped against England and Scotland Candidate Quiet Area criteria.

	L_day_ <55 dB(A)	> 9 hectares	(Perceived) Quiet	Relatively Quiet	(Perceived) Tranquil	Health and Wellbeing Benefits
**Garden**	✓		✓	✓	✓	✓
**Park**			✓		✓	✓
**Square**						✓

**Table 12:** Study sites mapped against alternative Candidate Quiet Area criteria.

	Perceived Natural	Perceived Built up	Perceived Calm	Accessible	Sounds Most Frequently Perceived	Sounds Least Frequently Perceived
**Garden**	✓	X	✓	✓	Nature	Dogs
**Park**	✓		✓	✓	Nature	People – Footsteps
**Square**			✓	✓	People - Vocal	Absence of sounds

## Data Availability

Data used in this paper is openly available from Heriot–Watt University archive system from 12th September 2019 when the project ends. Any use of the data must include attributions to the paper authors who collected the data. It can be found at https://doi.org/10.17861/3ca48e2d-7a6e-4553-ac3c-3b08a6612d19.
